# Successful Conservative Management of Thyroid Rupture and Haematoma Following Blunt Cervical Trauma: A Case Report

**DOI:** 10.7759/cureus.78368

**Published:** 2025-02-02

**Authors:** Chrysovalantis Stylianou, Ioannis Gogoulis, Vaia Karapepera, Konstantinos Chaidas, Michael Karanikas

**Affiliations:** 1 Radiology and Interventional Radiology Department, University General Hospital of Alexandroupolis, Alexandroupolis, GRC; 2 1st General Surgery Department, University General Hospital of Alexandroupolis, Alexandroupolis, GRC; 3 Ear, Nose, and Throat Clinic, General Hospital of Ioannina G. Hatzikosta, Ioannina, GRC; 4 Ear, Nose, and Throat Department, School of Medicine, Democritus University of Thrace, Alexandroupolis, GRC; 5 Ear, Nose, and Throat Department, University General Hospital of Alexandroupolis, Alexandroupolis, GRC; 6 General Surgery Department, Democritus University of Thrace, Alexandroupolis, GRC

**Keywords:** blunt cervical trauma, thyroid gland haematoma, thyroid storm, thyroid surgery, ultrasound thyroid

## Abstract

Parenchymal organ rupture is a serious emergency condition most commonly occurring in abdominal or thoracic viscera, whereas blunt neck trauma affecting the thyroid is rarely documented. We present the case of a 43-year-old male who sustained a blunt cervical trauma and presented with neck haematoma, respiratory discomfort, and hoarseness. The diagnosis of thyroid gland rupture of the left lobe was made via ultrasonography and computed sonography scans. The patient was admitted to the hospital for close monitoring and was managed conservatively. The patient recovered uneventfully and was discharged in good health after seven days.

## Introduction

Thyroid rupture following blunt cervical trauma is a rare but serious medical entity, with a limited number of cases presented in the English literature [1,2]. There is no proven predilection for rupture in either goitrous or normal glands; however, it has been proven to be a life-threatening condition. Specifically, it can lead to a condition described as a "thyroid storm" and the formation of large neck haematomas that can cause compressing phenomena to the trachea or vocal cord paralysis, resulting in acute respiratory distress. Therefore, early recognition and diagnosis and timely management are considered crucial in order to prevent serious life-threatening complications [3].

Herein, we present a case of unilateral blunt cervical trauma that led to thyroid haemorrhage, which was managed successfully with conservative treatment.

## Case presentation

**Figure 1:**
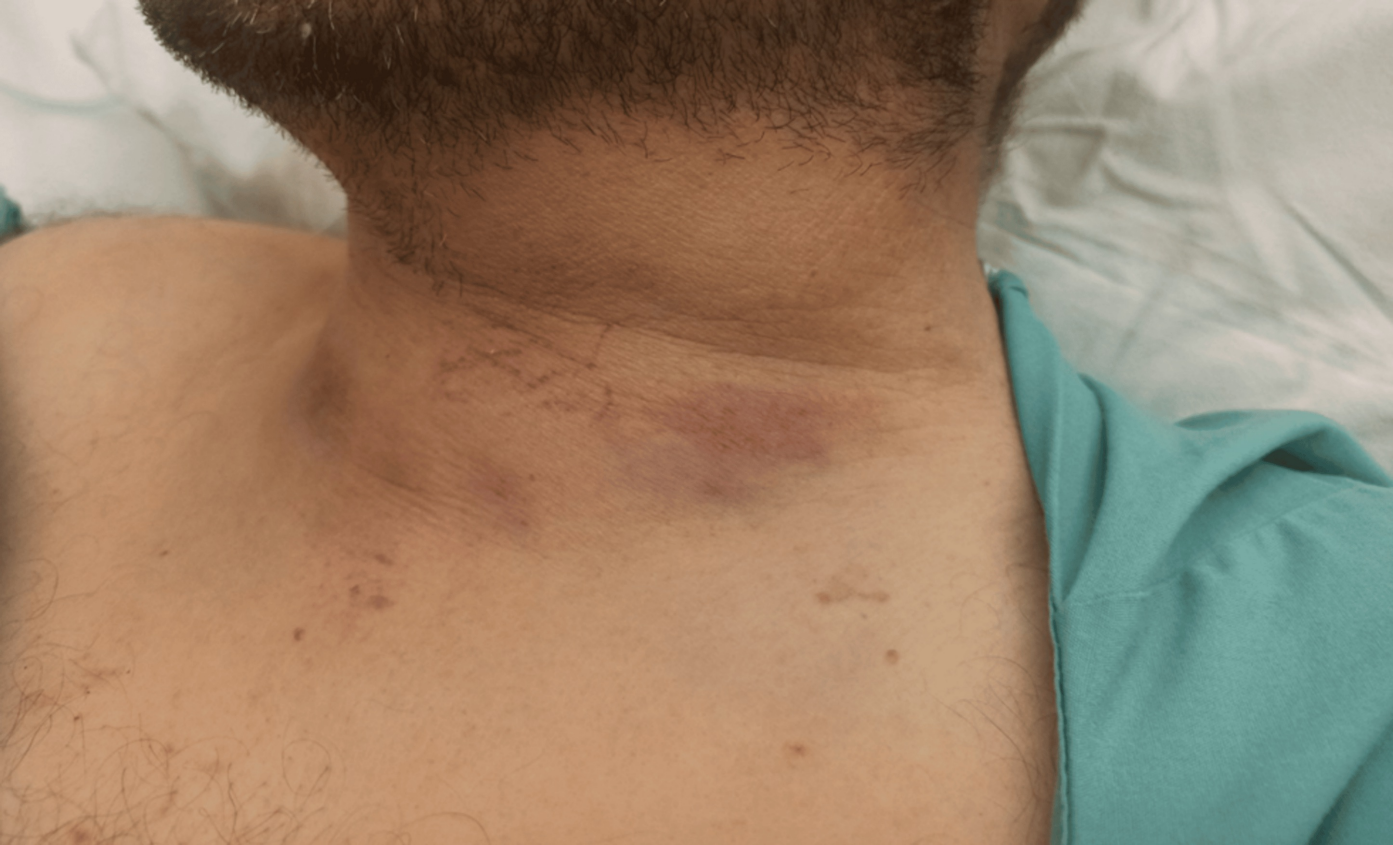
Left-sided oedema with ecchymosis

A 43-year-old male patient presented to the emergency department of our institution following a blunt anterior cervical trauma caused by a falling heavy object two hours prior. The patient complained of left-sided neck pain, acute onset of shortness of breath, and hoarseness. Clinical examination revealed left-sided neck oedema with ecchymosis, accompanied by tenderness on palpation (Figure 1).

There were no signs of emphysema. Vital signs included a blood pressure of 145/75 mmHg, a heart rate of 74 beats per minute, a respiratory rate of 14 breaths per minute, and an oxygen saturation of 98%. There was no past medical history.

After initial resuscitation and cervical immobilization, the patient was placed in an anti-Trendelenburg position in order to reduce neck oedema. Fibreoptic laryngoscopy revealed reduced left vocal cord and arytenoid cartilage mobility without signs of internal bleeding or airway obstruction.

**Figure 2:**
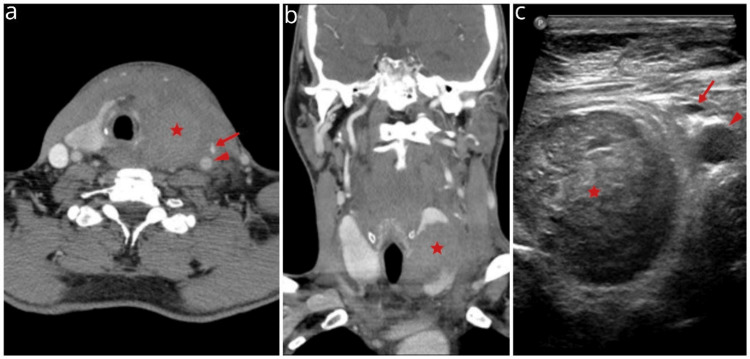
Initial contrast-enhanced computed tomography (CECT) (a, b) and 2D B-mode ultrasound (c) The images demonstrate parenchymal rupture and haematoma (star) of the left thyroid lobe. A narrowed left internal jugular vein (arrow) and displaced left common carotid artery (arrowhead) are also observed.

A contrast-enhanced computed tomography (CECT) scan of the cervical and thoracic region, with an angiography protocol and a parenchymal phase, indicated the presence of intraparenchymal haematoma of the left thyroid lobe measuring 48 mm both transversely and craniocaudally (Figures 2a, 2b). The cervical soft tissues contained diffuse haemorrhagic collections that extended caudally to the paratracheal region and the anterior mediastinum. There was a considerable deviation of the trachea and larynx, while the left internal jugular vein seemed narrowed; however, no sight of active contrast extravasation was found in the thyroid parenchyma or the surrounding tissues after a late scan at two minutes post-contrast. A complementary ultrasonography (US) examination confirmed vessel patency and haematoma volume stability (Figure 2c).

**Figure 3:**
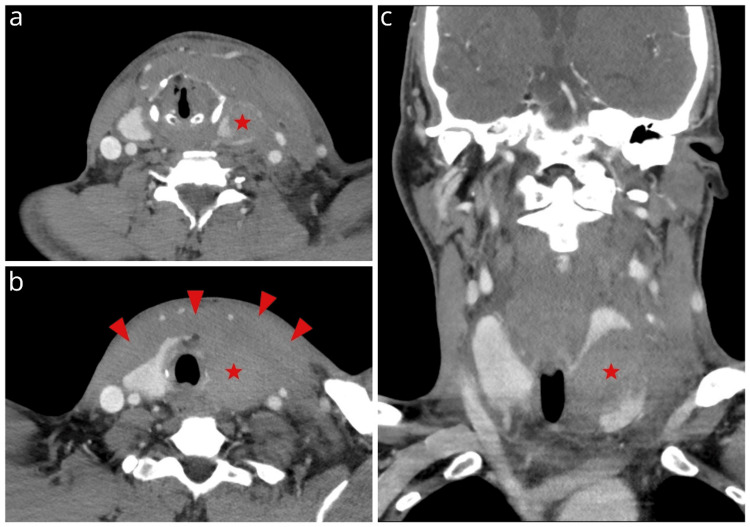
Three-day contrast-enhanced computed tomography (CECT) follow-up with axial images (a, b) and coronal reconstruction (c) The images indicate the stability of the haematoma size (star) and diffuse hyperdense haemorrhagic infiltration of the superficial lower cervical structures (arrowheads).

The patient was subsequently admitted to the surgical department, and a decision for conservative management was made in accordance with the American Association for the Surgery of Trauma (AAST) guidelines [4]. The patient was under close supervision, and respiratory function, including oxygen saturation and partial pressure of oxygen (pO_2_) levels, was continuously monitored for the first 24 hours. Intubation equipment and a tracheostomy kit were at the bedside to secure the patient's airway if needed. Blood samples were taken regularly to assess the thyroid function profile and prevent a thyroid storm. Free thyroxine (FT4) and free triiodothyronine (FT3) levels were elevated at FT4=2.0 ng/dL (reference range 0.7-1.8) and FT3=4.9 pg/mL (reference range: 2.3-4.1). Thyrotropin levels were decreased at TSH=0.03 mIU/L (reference range: 0.3-4.5). Furthermore, daily fibreoptic laryngoscopy was performed to ensure no airway compromise and evaluate vocal cord mobility. A repeat cervical CT scan was performed three days after the incident, indicating significant thyroid imaging improvement with no further expansion of the haematoma (Figure 3). Hoarseness resolved five days after trauma with normal vocal cord movement on endoscopy. The patient recovered uneventfully and was discharged seven days post-trauma.

**Figure 4:**
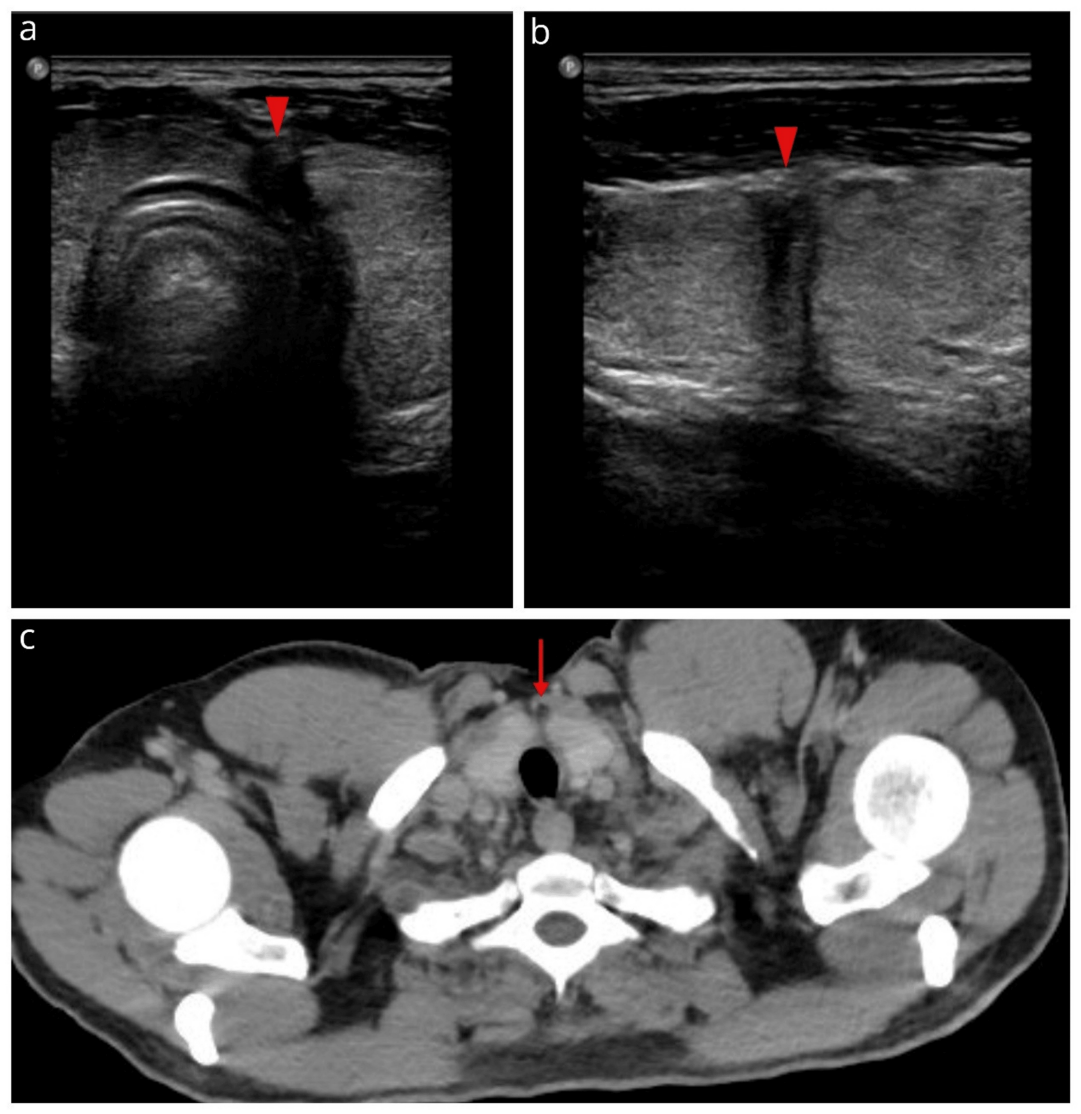
Two-month 2D B-mode ultrasound with transverse (a) and sagittal (b) views of the left thyroid lobe and an 18-month chest contrast-enhanced computed tomography (CECT) examination (c) Fibrous tissue is located at the level of the parenchymal rupture (arrowheads). CECT shows the parenchymal scar (arrow).

A follow-up assessment with US was performed seven days and two months after trauma, revealing fibrous tissue at the haematoma site, while the rest of the cervical structures were normal (Figures 4a, 4b). An incidental chest CECT 18 months later interestingly showed a thyroid scar paramedially (Figure 4c). Thyroid function was normalized (FT3: 1.8 pg/mL, FT4: 1.1 ng/dL, and TSH: 1.67 mIU/L).

## Discussion

The most common mechanism of traumatic thyroid gland rupture is high-energy traffic accidents with direct cervical injury. In contrast, trauma and assault are considered relatively rare causes. Although most patients develop symptoms immediately after trauma, a vast number of cases report late onset, which may vary from a few hours to even more than one day later [3].

A large number of studies have stated the importance of assessing patients' hormonal profiles for signs of thyroid toxicity, which can be subclinical. Specifically, thyroid storm symptoms are caused by the rupture of acini in the damaged thyroid tissue, which results in the subsequent release of hormones into the bloodstream and, thus, increased T4 levels. Symptoms accompanying this condition include hypertension, confusion, elevated temperature, and even death [5].

Immediate diagnosis and management are highly important in cases of neck trauma with suspected thyroid injury. Previous studies have proposed certain classifications and algorithms for the diagnosis and management of blunt thyroid injuries, where the type of treatment depends mostly on clinical symptoms and signs (Table 1). Emergency intubation and neck exploration are recommended in cases of large neck haematomas with adjacent structure compression, where the airway is at risk. On the other hand, some studies suggest conservative treatment in patients with no active haemorrhage or haematoma progression [6,7].

**Table 1:** Classification of blunt thyroid injuries as proposed by Heizmann et al. [8] and modified by Ramly et al. [9] CECT: contrast-enhanced computed tomography

Grading	Lesions	Management (According to CECT Findings and Preexisting Thyroid Abnormalities)
I	Small parenchymal laceration, bleeding nodules, subcapsular haematoma.	Conservative: close surveillance, daily sonographic follow-up. Non-conservative if there is an increase in haematoma size or patient haemodynamic instability.
II	Thyroid gland rupture ± parathyroidal haematoma.	
III	Thyroid gland rupture with significant cervical haematoma, including tracheal compression.	Non-conservative: emergency intubation, neck exploration.
IV	Thyroid gland rupture and cervical haematoma with associated lacerations to the larynx skeleton and/or to carotid and jugular vessels.	

Our case included a grade III thyroid rupture (parenchymal rupture, significant neck haematoma, and tracheal deviation) in which we pursued a conservative approach since the patient was haemodynamically and respiratory stable, did not use anticoagulation therapy, and CECT excluded major active contrast extravasation. During our initial approach and later observation, airway stability and vital signs were monitored constantly.

## Conclusions

We report a case of thyroid gland rupture after a blunt cervical trauma following a work accident. Conservative management is still a choice in patients with large haematomas and compression phenomena in the absence of respiratory discomfort and predisposing factors for haemorrhage expansion, such as anticoagulation therapy. However, this approach requires close monitoring and observation, a tracheostomy kit available at the bedside, and a highly trained and experienced trauma surgical team to take action in the onset of acute respiratory distress or thyroid storm.

## References

[1] Oka Y, Nishijima J, Azuma T (2007). Blunt thyroid trauma with acute hemorrhage and respiratory distress. J Emerg Med.

[2] Blaivas M, Hom DB, Younger JG (1999). Thyroid gland hematoma after blunt cervical trauma. Am J Emerg Med.

[3] Chartier LB, Turner JP (2010). Delayed intrathyroidal hematoma causing respiratory distress after a seemingly benign fall: a case report. Int J Emerg Med.

[4] Moore EE, Malangoni MA, Cogbill TH, Peterson NE, Champion HR, Jurkovich GJ, Shackford SR (1996). Organ injury scaling VII: cervical vascular, peripheral vascular, adrenal, penis, testis, and scrotum. J Trauma.

[5] Delikoukos S, Mantzos F (2007). Thyroid storm induced by blunt thyroid gland trauma. Am Surg.

[6] Sow YL, Aziz NA, Ng KL (2013). Thyroid rupture secondary to blunt neck trauma. Am J Emerg Med.

[7] Bansil NH (2021). Shattered thyroid gland from blunt neck trauma in a child. Am J Emerg Med.

[8] Heizmann O, Schmid R, Oertli D (2006). Blunt injury to the thyroid gland: proposed classification and treatment algorithm. J Trauma.

[9] Ramly N, Fadaee N, Aziz F, Azimi F (2020). Successful non-operative management of a traumatic thyroid haematoma in a goitrous gland. Trauma Case Rep.

